# Genetic diversity of *Aedes aegypti* and *Aedes albopictus* from cohabiting fields in Hainan Island and the Leizhou Peninsula, China

**DOI:** 10.1186/s13071-023-05936-5

**Published:** 2023-09-08

**Authors:** Minghui Zhao, Xin Ran, Yu Bai, Zu Ma, Jian Gao, Dan Xing, Chunxiao Li, Xiaoxia Guo, Xianyi Jian, Wei Liu, Yun Liao, Kan Chen, Hengduan Zhang, Tongyan Zhao

**Affiliations:** 1 State Key Laboratory of Pathogen and Biosecurity, Beijing Institute of Microbiology and Epidemiology, Beijing, China; 2 Jiangxi International Travel Healthcare Center, Nanchang, China; 3 Jiangxi Provincial Center for Disease Control and Prevention, Nanchang, China; 4 Jiangsu Provincial Center for Disease Control and Prevention, Nanjing, China

**Keywords:** *Aedes aegypti*, *Aedes albopictus*, Genetic diversity, Microsatellite DNA, *coxI*

## Abstract

**Background:**

*Aedes aegypti* and *Ae. albopictus* are important human arbovirus vectors that can spread arboviral diseases such as yellow fever, dengue, chikungunya and Zika. These two mosquito species coexist on Hainan Island and the Leizhou Peninsula in China. Over the past 40 years, the distribution of *Ae. albopictus* in these areas has gradually expanded, while *Ae. aegypti* has declined sharply. Monitoring their genetic diversity and diffusion could help to explain the genetic influence behind this phenomenon and became key to controlling the epidemic of arboviruses.

**Methods:**

To better understand the genetic diversity and differentiation of these two mosquitoes, the possible cohabiting areas on Hainan Island and the Leizhou Peninsula were searched between July and October 2021, and five populations were collected. Respectively nine and 11 microsatellite loci were used for population genetic analysis of *Ae. aegypti* and *Ae. albopictus*. In addition, the mitochondrial *coxI* gene was also selected for analysis of both mosquito species.

**Results:**

The results showed that the mean diversity index (PIC and SI values) of *Ae. albopictus* (mean PIC = 0.754 and SI = 1.698) was higher than that of *Ae. aegypti* (mean PIC = 0.624 and SI = 1.264). The same results were also observed for the *coxI* gene: the genetic diversity of all populations of *Ae. albopictus* was higher than that of *Ae. aegypti* (total *H* = 45 and Hd = 0.89958 vs. total *H* = 23 and Hd = 0.76495, respectively). UPGMA dendrogram, DAPC and STRUCTURE analyses showed that *Ae. aegypti* populations were divided into three clusters and *Ae. albopictus* populations into two. The Mantel test indicated a significant positive correlation between genetic distance and geographic distance for the *Ae. aegypti* populations (*R*^2^ = 0.0611, *P* = 0.001), but the correlation was not significant for *Ae. albopictus* populations (*R*^2^ = 0.0011, *P* = 0.250).

**Conclusions:**

The population genetic diversity of *Ae. albopictus* in Hainan Island and the Leizhou Peninsula was higher than that of *Ae. aegypti*. In terms of future vector control, the most important and effective measure was to control the spread of *Ae. albopictus* and monitor the population genetic dynamics of *Ae. aegypti* on Hainan Island and the Leizhou Peninsula, which could theoretically support the further elimination of *Ae. aegypti* in China.

**Graphical Abstract:**

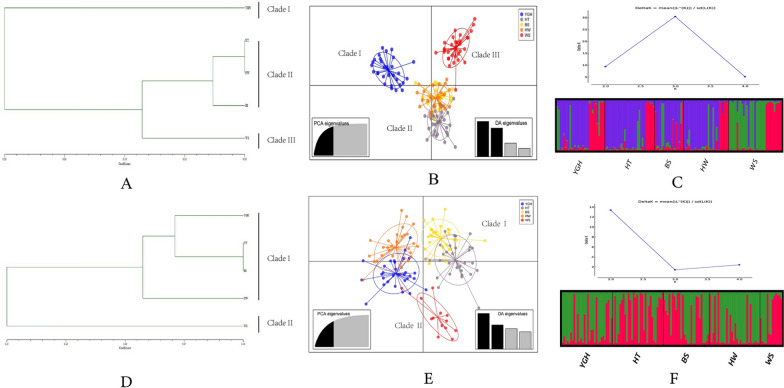

**Supplementary Information:**

The online version contains supplementary material available at 10.1186/s13071-023-05936-5.

## Background

*Aedes*-borne viruses (arboviruses) have always been a major global health concern. Dengue, yellow fever, chikungunya and Zika threaten approximately 3.9 billion people living in tropical and subtropical areas [1]. *Aedes aegypti* and *Ae. albopictus* are the main vectors of these arboviruses worldwide, and their high ecological and physiological plasticity has facilitated their current global distribution [2]. *Aedes aegypti* originated in Africa, and its distribution in China has been limited to a few provinces, including Hainan Island and the Leizhou Peninsula in Guangdong Province [3]. However, *Ae. albopictus*, which originated in Asia and is also known as the Asian tiger mosquito, has spread to all continents except for Antarctica and is considered the most invasive mosquito species [4, 5]. *Aedes albopictus* is widespread in China, extending to Liaoning Province in the north, Gansu Province in the northwest and Hainan Province in the south [6].

The two mosquito species coexist on Hainan Island and the Leizhou Peninsula, the main dengue epidemic areas at the southernmost tip of mainland China. There were two dengue epidemics on Hainan Island at the end of the twentieth century and one on Leizhou Peninsula at the beginning of the twenty-first century. *Aedes aegypti* was the vector of transmission in these outbreaks [7–10]. More recently, however, *Ae. albopictus* has become the main vector, and the breeding areas of *Ae. aegypti* on these two islands have gradually decreased over the last 40 years. Guangxi was the first province in China to declare *Ae. aegypti* eradication. Between 1986 and 1991, Guangxi carried out comprehensive control of *Ae. aegypti* by application of insecticides, fish culture in water jars and other methods and achieved the goal of eliminating *Ae. aegypti* from the entire province in just 6 years [11]. *Aedes aegypti* was not found during subsequent monitoring, but *Ae. albopictus* densities were very high [12]. Various explanations have been proposed for the displacement of *Ae. aegypti* by *Ae. albopictus*, including interspecific competition [13], satyrization [14–16] and differential adaptability to environmental changes [17].

Genetic diversity and population structure are fundamental to understanding population dynamics and dispersal [18]. Genetic structure and variation are related to many factors, including vector control campaigns, levels of urbanization, trade flows between cities and the number of colonization events [19]. There are some reports on the genetic characteristics of *Ae. aegypti* and *Ae. albopictus* populations in China. For *Ae. aegypti*, most of the research has focused on the genetic characteristics of invaded *Ae. aegypti* populations in Yunnan Province [3, 20–23]. For *Ae. albopictus*, much research has been done on its genetic diversity and population structure in different areas of China at different scales. At the national level, some studies have shown that continuous dispersal supported by human activities has contributed to strong gene flow, inhibiting population differentiation and promoting genetic diversity among *Ae. albopictus* populations, and that climatic factors may also influence genetic diversity. [24–29]. There are also many provincial reports on the genetic diversity of *Ae. albopictus* populations in different regions of China, such as Guangdong, Zhejiang, Fujian and Hunan Provinces [30–33], which all suggest low differentiation of *Ae. albopictus* populations. At the regional level, the rapid expansion of high-speed railways, air routes and highways has accelerated the dispersal of mosquitoes in the Yangtze River basin, inhibiting population differentiation and promoting genetic diversity among *Ae. albopictus* [34]. However, in their cohabiting areas such as Hainan Island and the Leizhou Peninsula, there were no reports on the genetic diversity of *Ae. aegypti*, or the different dispersal pattern with *Ae. albopictus* in the sympatric areas, especially the genetic reasons for the change in population size of these two species in the past 40 years.

In Vietnam, a high degree of genetic polymorphism was found in invasive *Ae. aegypti* mosquitoes. Individual abundance of *Ae. aegypti* and *Ae. albopictus* was also influenced by climate and habitat in the sympatric region [2]. In Penang, *Ae. albopictus* was found in most areas, and it was postulated that the species is beginning to replace *Ae. aegypti* and may become the primary vector of dengue virus [35]. In Central Africa, *Ae. albopictus* is the dominant species in most urban areas located below 6°N where it tends to replace the native *Ae. aegypti* [36]. On the other hand, it was found that the coexistence of *Ae. albopictus* and *Ae. aegypti* in the same geographical areas may increase the risk of infection or co-infection for humans, especially during outbreaks or arboviral expansions [1]. Most studies focus on monitoring the densities of these two mosquito species or conduct population genetics research on a single species in China. However, there is no report on the difference in genetic diversity and dispersal patterns between *Ae. albopictus* and *Ae. aegypti* in cohabiting fields, especially based on the different shift in distribution and densities of two species.

In the present study, the genetic diversity of *Ae. aegypti* and *Ae. albopictus* collected from the cohabiting areas of Hainan Island and the Leizhou Peninsula was assessed, based on microsatellite molecular markers in DNA (SSR) and mitochondrial DNA marker (*coxI*). Understanding the population genetic dynamics between these two mosquitoes could help to understand why the distribution of *Ae. aegypti* has decreased significantly while the distribution of *Ae. albopictus* has increased on Hainan Island and the Leizhou Peninsula over the last 40 years. On the other hand, it also has important implications for vector control strategies in China, especially in the plan to eliminate *Ae. aegypti* on the Leizhou Peninsula and further reduce *Ae. aegypti* on Hainan Island.

## Methods

### Sample collection and DNA extraction

**Fig. 1:**
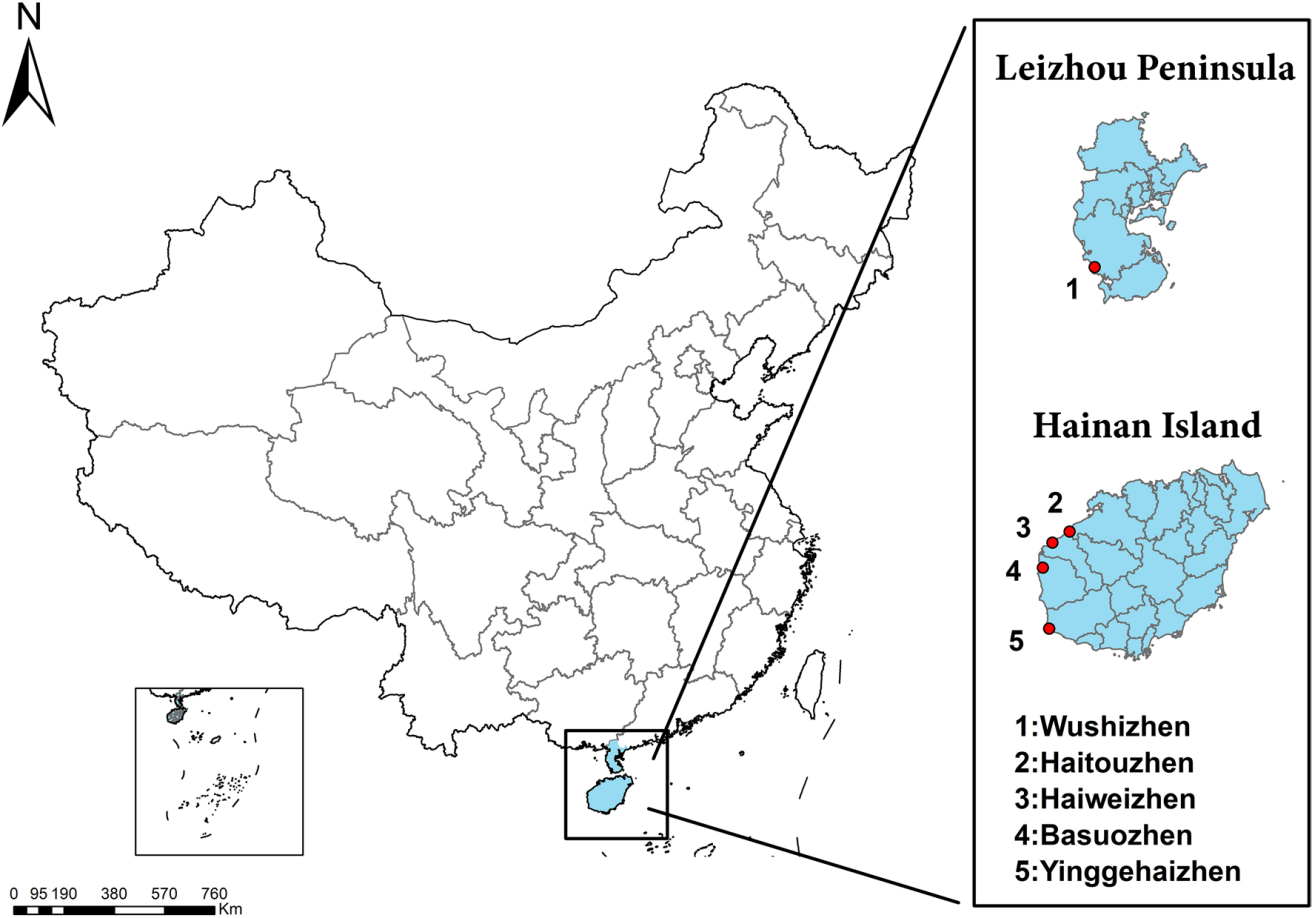
Sampling map of *Aedes aegypti* and *Ae. albopictus* populations in Hainan Island and the Leizhou Peninsula, China

Larvae, pupae and adult *Ae. aegypti* and *Ae. albopictus* were collected from five cohabiting areas in Hainan Island and the Leizhou Peninsula from July to October 2021 (Table 1 and Fig. 1). All the developmental stages of mosquitoes were brought back to a field laboratory. The larvae and pupae were reared until adult mosquitoes emerged. The species of all adult mosquitoes were identified based on morphology [6]. Adult mosquitoes were stored in sterile tubes containing anhydrous ethanol prior to DNA extraction. Whole genomic DNA was extracted individually from each mosquito using a DNeasy^®^ Blood & Tissue Kit (Qiagen, Hilden, Germany) according to the manufacturer’s standard protocol, and extracted DNA was stored at − 20 °C prior to subsequent experiments.

**Table 1:** Sampling locations and number of *Aedes aegypti and Ae. albopictus*, respectively

Localities	*Ae.aegypti* (N*)		Total (N*)	*Ae.albopictus* (N*)		Total (N*)
	Female	Male		Female	Male	
Leizhou Peninsula						
Wushizhen (WS)	20(15)	20(18)	40(33)	7(7)	18(6)	25(13)
Hainan Island						
Haiweizhen (HW)	15(12)	22(16)	37(28)	15(15)	15(15)	30(30)
Haitouzhen (HT)	18(17)	18(14)	36(31)	15(15)	15(15)	30(30)
Basuozhen (BS)	18(11)	15(7)	33(18)	15(12)	15(15)	30(27)
Yinggehaizhen (YGH)	20(16)	20(14)	40(30)	15(15)	15(15)	30(30)
Total (N*)	91(71)	95(69)	186(140)	67(64)	78(66)	145(130)

^*^Total number (number used for molecular experiments)

### Microsatellite DNA genotyping and data analysis

Nine microsatellite loci (AT1, AG2, AG7, AC2, AC7, B07, F06, SQM6 and SQM7) and 11 microsatellite loci (BW-P1, BW-P3, BW-P6, BW-P18, BW-P22, BW-P23, BW-P24, BW-P26, BW-P27, BW-P35 and BW-P36) (Additional file 1: Table S1) were selected for amplification by PCR using fluorescence-labeled primers for *Ae. aegypti* and *Ae. albopictus,* respectively, based on previous research [3, 28, 37]. Amplified fragments were separated by capillary electrophoresis, and the microsatellite DNA fragments were entered into Excel for analysis. GenAlex v.6.5 was used to estimate the pairwise genetic relatedness between individuals by the LRM estimator. The coefficient of relatedness *r* ≥ 0.25 was used to define a full sib relationship (parents and offspring, or sibs sharing the same parents) [38, 39]. To reduce the sibling bias within populations for genetic structure analysis, only one individual was selected from each putative full-sibling group within each population. In addition, genetic diversity within each cohabiting area was estimated using the average number of alleles (Na), effective number of alleles (Ne), Shannon’s diversity index (SI), observed heterozygosity (Ho), expected heterozygosity (He) and interpopulation variation assessed by AMOVA using GenAlEx v.6.5 [39]. The DAPC analysis was performed on the R platform using the “Adegenet” data analysis module and mobilizing the “inbreeding and seppop” commands to test the normality of inbreeding coefficients for different populations [40]. In addition, population genetic structure was assessed in STRUCTURE 2.3.4 software, and optimal K values were calculated using the ΔK method [41, 42], with the best K value calculated using Structure Harvester, https://taylor0.biology.ucla.edu/structureHarvester/. The output data were subjected to 1000 iterations using the greedy algorithm of CLUMPP v.1.1.2 [43] and finally visualized and analyzed using DISTRUCT v.1.1 [44]. Polymorphic information content (PIC) was assessed using the Microsatellite Toolkit [45]. The null allele frequency of each locus, deviation from Hardy-Weinberg equilibrium (HWE) and linkage disequilibrium (LD) were calculated using GENEPOP version 4.1.4 [46]. To detect bottlenecks, the SIGN test for heterozygosity excess was performed with TPM in BOTTLENECK v.1.2.02 [47]. In addition, NTSYS v2.10e was used to plot the UPGMA tree based on Nei's genetic distance, and GenAlex v.6.5 was used for the genetic correlation test (Mantel test).

### Mitochondrial DNA sequencing and phylogenetic analysis

The whole mitochondrial *coxI* gene of *Aedes* mosquitoes was amplified using the following primer pairs: *coxI*-F: 5′GGTCAACAAATCATAAAGATATTGG3′ and *coxI*-R: 5′ TCCAATGCACTAATCTGCCATATTA3′ [48, 49]. The 25 µl reaction mixtures contained 12.5 µl PCR Mix (TaKaRa, Japan), 8.5 µl ddH_2_O (TaKaRa, Japan), 1 µl forward primers and 1 µl reverse primers (synthesized by Sangon Biotech), with 2 µl DNA template. The PCR amplification consisted of a 5-min pre-denaturation at 94 °C followed by 35 cycles of 94 °C for 30 s, 60 for 45 s and 72 °C for 60 s, with a final elongation at 72 °C for 5 min, using the BIORAD (USA) thermal cycler. All the PCR products were detected and separated by 2% agarose gel electrophoresis, and the positive PCR products were bidirectionally sequenced by Beijing Genomics Institution (BGI), China. The sequences were checked by the BioEdit and alignmented by cluster W, and the mutation sites were determined. Sequences were sorted in Mega v7 for subsequent data analysis. The base content and polymorphism were analyzed by Mega v7 [50]. Haplotype index (number of haplotypes-H, haplotype diversity-Hd, nucleotide diversity-π, average number of nucleotide differences-k) and mismatch distribution analysis of *Aedes* populations were calculated by DnaSP v6.12.03 [51], and the haplotype network diagram was plotted by Popart v1.7 [52]. Molecular variance (AMOVA), neutrality test (Tajima’s D and Fu’s Fs) and Fst test with the calculation Nm = (1/Fst + 1)/4 were calculated in ARLEQUIN v3.1 [53].

## Results

### Sampling data and species identification

Possible breeding sites of *Ae. aegypti* on Hainan Island and the Leizhou Peninsula from July to October 2021 were searched and surveyed, and the results were consistent with previous reports stating that the breeding sites specific for *Ae. aegypti* have been greatly reduced [10, 54–56]. Only five populations co-habiting with *Ae. albopictus* were collected, one from the Leizhou Peninsula and four from Hainan Island (Table 1). In contrast, *Ae. albopictus* was present in all sites searched. Species identification was performed 3 days after the emergence of adult mosquitoes. We collected both female and male mosquitoes because we believe that the study of genetic diversity should include both sexes, consistent with the approach of other studies [57].

### Genetic diversity based on microsatellite DNA

We excluded 46 *Ae. aegypti* and 15 *Ae. albopictus* samples of full siblings at collection sites, and the remaining 140 *Ae. aegypti* and 130 *Ae. albopictus* samples were used for further molecular analysis (Table 1). The pairwise genetic relatedness of all samples within and between populations was compared using the LRM estimator (Additional file 2: Table S2). *Aedes aegypti* had a greater number of within-population comparisons (total percentage 1.06%) than *Ae. albopictus* (0.64%).

**Fig. 2:**
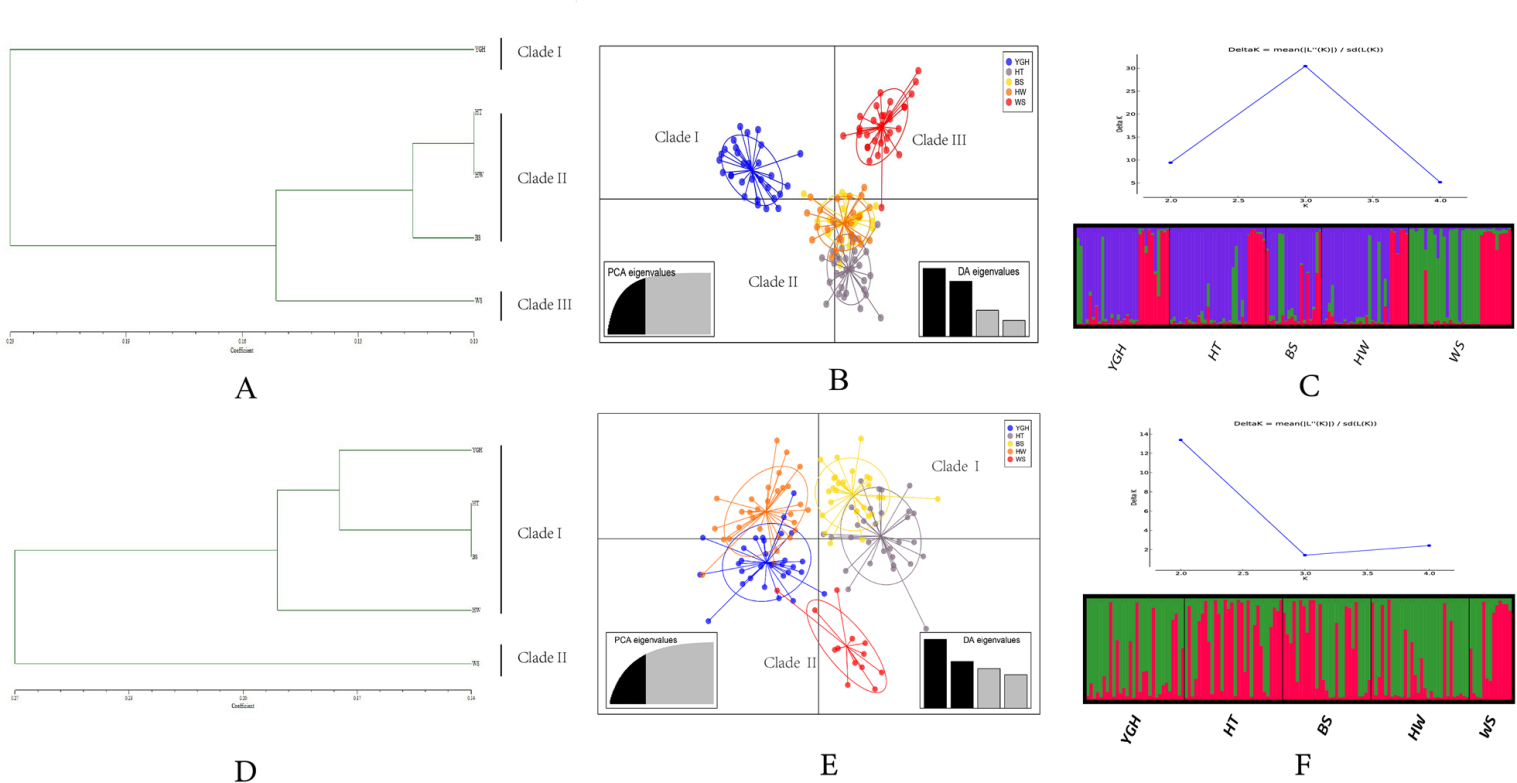
Population structure analysis based on SSR for *Aedes aegypti* (**A**–**C**) and *Ae. albopictus* (**D**–**F**). **A** and **D**: UPGMA dendrogram based on Nei's genetic distance; **B** and **E**: DAPC analysis. **C** and **F**: ΔK values and STRUCTURE bar plots

The genetic diversity of the nine microsatellite loci for *Ae. aegypti* and the 11 microsatellite loci for *Ae. albopictus* are shown in Table 2. The Na, PIC and SI values of each locus were consistent, indicating that most loci were polymorphic in both *Aedes* populations [27, 28]. The diversity index of *Ae. albopictus* was higher than that of *Ae. aegypti*. The results of null allele evaluation indicated that loci F06, BW-P1 and BW-P27 had a high probability of null alleles (> 0.2) [58–60]. The mean value of observed heterozygosity (Ho) was lower than the expected heterozygosity (He) in all the *Aedes* populations. A test of Hardy-Weinberg equilibrium (HWE) at each locus per population indicated that all ten populations were significantly departed from HWE (Additional file 3: Table S3). Significant results for the linkage disequilibrium (LD) test were obtained for 31 out of 180 pairs (17.2%) in *Ae. aegypti* and 55 out of 275 pairs (20.0%) in *Ae. albopictus*. The TPM model was used to assess the recent population bottleneck and showed a significant difference (*P* < 0.05) in the BS population of *Ae. aegypti* (Table 3). Analysis of molecular variance (AMOVA) showed that most of the variation occurred within the populations, with percentages of variation of 90.7% for *Ae. aegypti* and 96.4% for *Ae. albopictus*, respectively (Additional file 4: Table S4). The results of UPGMA cluster analysis were shown in Fig. 2A, D, which indicated that *Ae. aegypti* populations were clustered into three branches and *Ae. albopictus* populations were clustered into two branches. In addition, the results of the DAPC analysis were similar to those of the cluster analysis (Fig. 2B, E). The STRUCTURE analysis also suggested that *Ae. aegypti* could be divided into three genetic clusters, while *Ae. albopictus* could be divided into two (Fig. 2C, F). The Mantel test showed a positive correlation between genetic distance and geographical distance (*R*^2^ = 0.0611, *P* = 0.001 for *Ae. aegypti* and *R*^2^ = 0.0011, *P* = 0.250 for *Ae. albopictus*).

**Table 2:** Genetic diversity indices for nine microsatellite loci of *Aedes aegypti* populations and 11 microsatellite loci of *Ae. albopictus* populations

Mosquito	Locus	Na	Ne	Null allele frequency	PIC	SI	Ho	He
*Ae. aegypti*	B07	9.200	3.863	0.073	0.740	1.675	0.626	0.718
	F06	1.800	1.231	0.267	0.160	0.272	0.035	0.163
	SQM6	6.400	3.702	0.041	0.731	1.462	0.702	0.702
	SQM7	5.800	2.733	0.039	0.665	1.193	0.512	0.575
	AT1	8.400	5.390	0.032	0.857	1.862	0.809	0.802
	AG2	6.200	2.959	0.044	0.665	1.288	0.564	0.630
	AG7	9.400	4.824	0.140	0.801	1.825	0.566	0.775
	AC2	4.000	2.833	0.032	0.612	1.139	0.640	0.633
	AC7	3.000	1.738	0.010	0.383	0.660	0.383	0.375
	Mean	6.022	3.253		0.624	1.264	0.537	0.597
*Ae. albopictus*	BW-P1	12.800	8.274	0.266	0.915	2.282	0.398	0.876
	BW-P3	3.800	2.254	0.155	0.479	0.928	0.306	0.555
	BW-P6	6.800	3.003	0.018	0.630	1.314	0.798	0.662
	BW-P18	5.400	2.891	0.051	0.626	1.261	0.616	0.641
	BW-P22	7.200	4.401	0.067	0.783	1.655	0.651	0.765
	BW-P23	13.600	9.280	0.138	0.918	2.310	0.648	0.871
	BW-P24	10.800	5.371	0.076	0.820	1.918	0.695	0.804
	BW-P26	9.000	3.992	0.000	0.751	1.699	0.784	0.739
	BW-P27	11.600	7.823	0.262	0.914	2.176	0.409	0.856
	BW-P35	7.000	3.474	0.095	0.705	1.475	0.546	0.677
	BW-P36	8.000	4.434	0.136	0.747	1.659	0.513	0.739
	Mean	8.727	5.018		0.754	1.698	0.579	0.744

**Table 3:** Bottleneck tests based on TPM model for *Aedes aegypti and Ae. albopictus* populations

Mosquito	TPM model	YGH	HT	BS	HW	WS
*Ae. aegypti*	He < Heq	3	4	7	5	3
	He > Heq	5	5	2	3	6
	P	0.54005	0.58148	0.03063^*^	0.18008	0.43521
*Ae. albopictus*	He < Heq	7	3	5	3	2
	He > Heq	4	8	6	8	9
	P	0.09819	0.28368	0.47455	0.28380	0.11056

^*^P < 0.05

### Genetic diversity based on the mitochondrial *coxI* gene

**Fig. 3:**
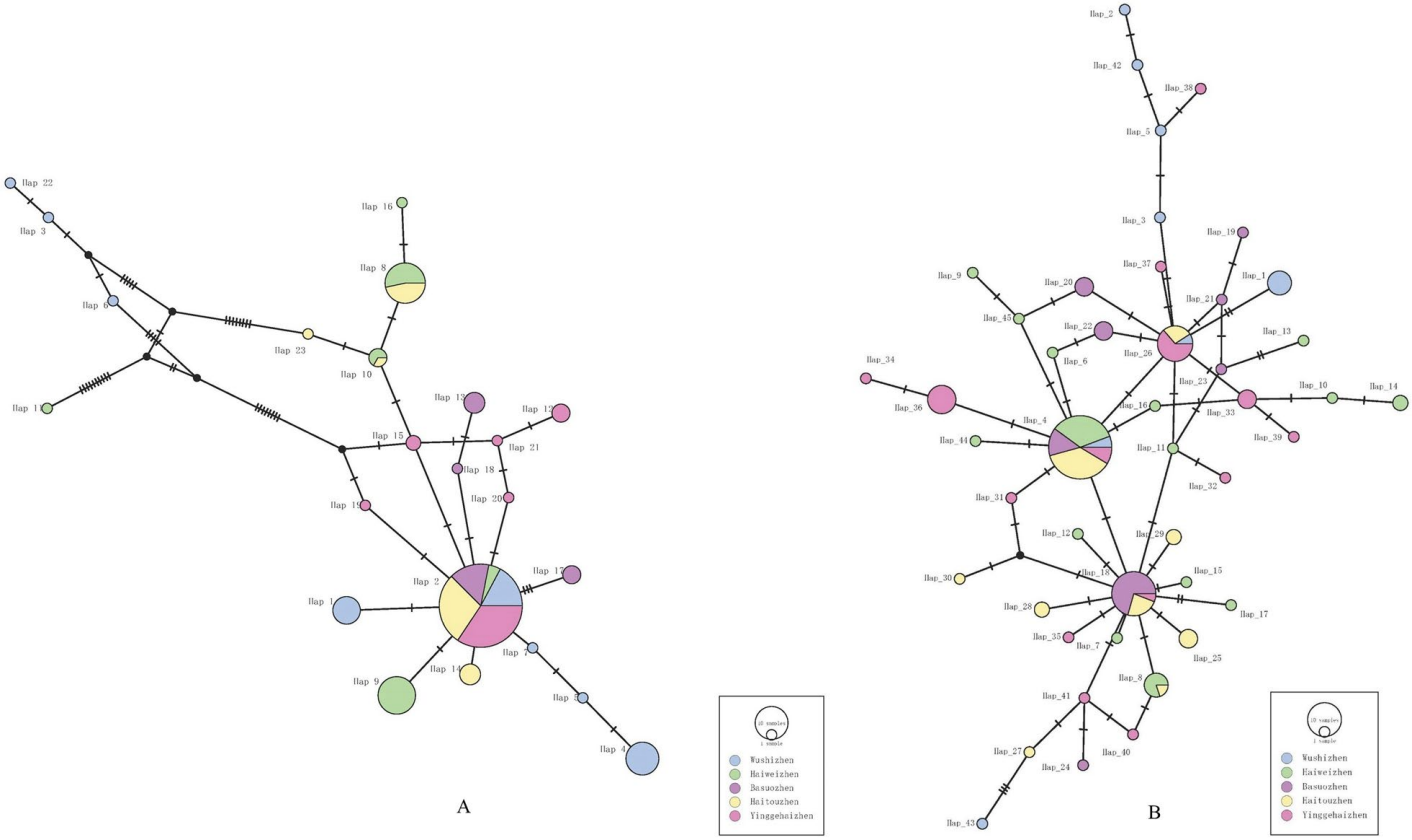
TCS network among haplotypes based on *coxI* gene for **A** *Aedes aegypti* and **B** *Ae. albopictus*. Each line segment represents a single mutation. The size of the circle represents the number of samples

Base polymorphism and haplotype diversity of the final 1330 bp *coxI* genes are shown in Table 4. The nucleotide composition was A + T rich for both *Aedes* species, consistent with the characteristics of insect mitochondrial DNA (accession numbers: OR144884-OR144906 for *Ae. aegypti* and OR144827-OR144871 for *Ae. albopictus*) [61]. High haplotype diversity was found in all the populations (Hd = 0.45977–0.77083 for *Ae. aegypti*, Hd = 0.77083–0.88966 for *Ae. albopictus*), except for the YGH population of *Ae. aegypti* (Hd = 0.45977), contrasting with low nucleotide diversity (*π* = 0.00066–0.00346 for *Ae. aegypti*, *π* = 0.00111–0.00262 for *Ae. albopictus*). A total of 23 haplotypes were recorded from 140 individuals of *Ae. aegypti* (Fig. 3). The WS population had the largest number of haplotypes (8), followed by the HW (6) and YGH (6) populations. In comparison, the number of haplotypes from *Ae. albopictus* was relatively higher. A total of 45 haplotypes were recorded from 130 individuals of *Ae. albopictus* (Fig. 3). The HW population had the largest number of haplotypes (15), followed by the YGH (14) and HT (9) populations. The two *Aedes* mosquito species in BS had the lowest number of haplotypes (4 and 8, respectively). There were three shared haplotypes and 20 unique haplotypes in *Ae. aegypti* populations with the dominant haplotypes being Hap_2. There were 4 shared haplotypes and 41 unique haplotypes in *Ae. albopictus* populations with the dominant haplotypes being Hap_4, Hap_18 and Hap_26.

**Table 4:** Base polymorphism and haplotype diversity of *Aedes aegypti* and *Ae. albopictus*

Mosquito	Population	*N*	(A + T) %	C	V	Pi	S	H	Hd	*π*	*k*	Haplotypes
*Ae. aegypti*	YGH	30	69.6	1326	4	3	1	6	0.45977	0.00066	0.87126	Hap_2(22), Hap_12(3), Hap_15(2), Hap_19(1), Hap_20(1), Hap_21(1)
	HT	31	69.5	1325	5	4	1	5	0.61290	0.00114	1.50968	Hap_2(18), Hap_8(7), Hap_10(1), Hap_14(4), Hap_23(1)
	BS	18	69.5	1325	5	5	0	4	0.64706	0.00126	1.67320	Hap_2(10), Hap_13(4), Hap_17(3), Hap_18(1)
	HW	28	69.5	1305	25	4	21	6	0.70899	0.00262	3.45767	Hap_2(3), Hap_8(8), Hap_9(13), Hap_10(2), Hap_11(1), Hap_16(1)
	WS	33	69.5	1308	22	21	1	8	0.77083	0.00346	4.56818	Hap_1(7), Hap_2(11), Hap_3(1), Hap_4(10), Hap_5(1), Hap_6(1), Hap_7(1), Hap_22(1)
	Total	140	69.5	1290	40	30	10	23	0.76495	0.00216	2.87338	
*Ae. albopictus*	YGH	30	70.0	1317	13	5	8	14	0.88966	0.00163	2.15862	Hap_4(3), Hap_18(1), Hap_26(7), Hap_31(1), Hap_32(1), Hap_33(3), Hap_34(1), Hap_35(1), Hap_36(7), Hap_37(1), Hap_38(1), Hap_39(1), Hap_40(1), Hap_41(1)
	HT	30	70.1	1320	10	5	5	9	0.78851	0.00111	1.47816	Hap_4(13), Hap_8(1), Hap_18(4), Hap_25(3), Hap_26(3), Hap_27(1), Hap_28(2), Hap_29(2), Hap_30(1)
	BS	27	70.1	1322	8	5	3	8	0.76638	0.00137	1.81766	Hap_4(5), Hap_18(12), Hap_19(1), Hap_20(3), Hap_21(1), Hap_22(3), Hap_23(1), Hap_24(1)
	HW	30	70.0	1312	18	7	11	15	0.83218	0.00181	2.40230	Hap_4(12), Hap_6(1), Hap_7(1), Hap_8(4), Hap_9(1), Hap_10(1), Hap_11(1), Hap_12(1), Hap_13(1), Hap_14(2), Hap_15(1), Hap_16(1), Hap_17(1), Hap_44(1), Hap_45(1)
	WS	13	69.9	1319	11	7	4	8	0.85897	0.00262	3.48718	Hap_1(5), Hap_2(1), Hap_3(1), Hap_4(2), Hap_5(1), Hap_26(1), Hap_42(1), Hap_43(1)
	Total	130	70.0	1294	36	22	14	45	0.89958	0.00176	2.34705	

C: Number of conserved sites; V: number of variable sites; Pi: number of parsimony information sites; S: number of singletons; H: number of haplotypes; Hd: haplotype diversity; π: nucleotide diversity; k: average number of nucleotide differences

In the neutrality test (Additional file 5: Table S5), Tajima’s D showed that only the HW *Ae. aegypti* and *Ae. albopictus* populations had experienced population expansion. A negative value of Fu’s FS for *Ae. aegypti* was obtained only for YGH, but the *P* values were > 0.05. In contrast, among the *Ae. albopictus* populations, three (YGH, HT and HW) had experienced population expansion.

**Fig. 4:**
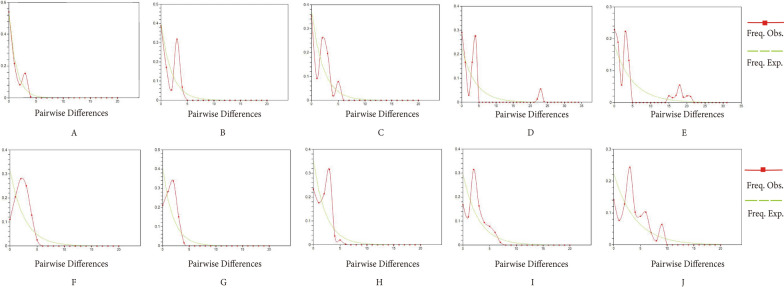
Mismatch distributions analysis for *coxI* gene of *Aedes aegypti* (**A**–**E**) and *Ae. albopictus* (**F**–**J**). **A**–**E** and **F**–**J** are YGH, HT, BS, HW and WS populations, respectively

AMOVA showed that most of the variation occurred within populations, with 84.6% and 87.8% percentage variations, respectively (Additional file 4: Table S4). The mismatch distribution analysis used to estimate the historical population dynamics is shown in Fig. 4 and indicated population expansion (unimodal) in the YGH and HT populations of *Ae. aegypti* and the YGH, HT and HW populations of *Ae. albopictus*.

Pairwise Fst values indicated that most pairs were significantly different (*P* < 0.05), except for the BS and HW, HT and HW population pairs of *Ae. albopictus*. The Nm calculated by Fst showed that most pairwise values were > 1, indicating frequent communication between most populations (Additional file 6: Table S6).

## Discussion

Hainan Island and the Leizhou Peninsula are cohabiting fields of *Ae. aegypti* and *Ae. albopictus* in China. *Aedes aegypti* used to be the main vector species of dengue; however, with the development of the economy and changes in the ecological environment, *Ae. albopictus* has become the main vector species in these areas [62–64]. Only five *Ae. aegypti* populations were sampled that were cohabiting with *Ae. albopictus*, whereas *Ae. albopictus* was ubiquitous in all researched areas. Some studies have reported that *Ae. aegypti* had a relatively short active range compared to other mosquitoes [65]. In recent years, the distribution of *Ae. aegypti* in these areas has become increasingly restricted, and they could only be found in a few fishing villages along the coast. The sharp decline in *Ae. aegypti* over the last 40 years is closely linked to the vector control measures and ecological reconstruction of these villages, which have greatly altered the mosquito breeding environment. However, the distribution of *Ae. albopictus* in these areas might have increased because of its strong environmental adaptability. The present study investigated the reasons for the decline of *Ae. aegypti* and the expansion of *Ae. albopictus* on Hainan Island and the Leizhou Peninsula based on population genetics studies.

### Genetic diversity

According to the microsatellite DNA results, the mean diversity index (PIC and SI values) of *Ae. albopictus* was higher than that of *Ae. aegypti*, indicating that the population diversity of *Ae. albopictus* was greater and it was better able to adapt environmental changes than populations of *Ae. aegypti*. Microsatellite DNA are widely used to evaluate genetic diversity and population structure because of the advantages of simple operation, easy detection and high polymorphism [66, 67]; the microsatellite DNA results in the present study showed that most loci were polymorphic [27, 28]. Mitochondrial *coxI* gene with strict maternal inheritance, conservative genetic structure and moderate evolution rate is also an effective molecular marker to study the genetic structure of mosquitoes [35, 68, 69]. The genetic diversity of all *Ae. albopictus* populations revealed by *coxI* gene was higher than that of *Ae. aegypti* in the present research. High haplotype number and haplotype diversity indicated that the population was more complex and better able to resist the effects of environmental change [70]. *Aedes albopictus* is a native mosquito species in China, and its adaptive evolution with the local environment might be longer than that of *Ae. aegypti*. Small and isolated populations are generally susceptible to negative factors such as environmental change, inbreeding and genetic randomness, which can reduce individual fitness and population viability, leading to reduced genetic diversity [71–73]. On Hainan Island and the Leizhou Peninsula, *Ae. aegypti* belongs to these small and range-restricted groups compared to *Ae. albopictus*. The population genetic results of this study reveal that *Ae. aegypti* populations with restricted range are less genetically diverse than widely distributed *Ae. albopictus* populations. Therefore, the elimination of breeding sites and the prevention of *Ae. aegypti* invasion should be continued to maintain the low genetic diversity of *Ae. aegypti* populations and to facilitate the complete elimination of *Ae. aegypti* from Hainan Island and the Leizhou Peninsula in the future.

### Haplotype distribution

The haplotype diversity of all populations of *Ae. albopictus* was higher than that of *Ae. aegypti.* There were three predominant haplotypes (Hap_4, Hap_18 and Hap_26) in *Ae. albopictus* populations on Hainan Island and the Leizhou Peninsula, which were also found in other areas of China [25, 28, 74]. However, only one predominant haplotype was recorded in *Ae. aegypti* populations, from which the other 22 haplotypes evolved. High haplotype diversity and low nucleotide diversity were recorded in all the *Aedes* populations except the YGH population of *Ae. aegypti*, which may indicate rapid population growth after a bottleneck period and may have been caused by the widespread use of pesticides and the impact of human activities [25, 75]. The YGH population of *Ae. aegypti* exhibited low haplotype diversity and a low nucleotide diversity model and may have undergone founder events in which a new population was established by a small number of individuals drawn from a large ancestral population; the results of the UPGMA, DAPC and STRUCTURE analysis seemed to support this view (Fig. 2) [35, 76].

### Population differentiation and genetic structure

Significant differentiation was found between all the *Ae. aegypti* populations, indicating less gene flow between populations and ultimately leading to a significant positive correlation between genetic distance and geographical distance [28, 32]. The degree of genetic differentiation varied between different populations of *Ae. albopictus*, with the WS population showing relatively high differentiation from other populations (Additional file 6: Table S6).

The results of both UPGMA and DAPC analysis supported the division of *Ae. aegypti* into three genetic clusters and *Ae. albopictus* into two, with the same population composition within each major genetic branch. Figure 2A, B shows that the YGH and WS populations of *Ae. aegypti* were genetically distant from the other three populations and formed two separate genetic clusters. The HT, HW and BS populations were genetically closer and formed a separate genetic cluster, which was also consistent with the geographical distance between the populations. Therefore, the genetic distance of *Ae. aegypti* populations was significantly and positively correlated with the geographic distance. Figure 2D, E shows that the *Ae. albopictus* population could be divided into two genetic clusters, with the WS population being genetically distant from the other four populations and forming a separate cluster. The YGH, HW, HT and BS populations formed one other large genetic cluster. The WS *Ae. albopictus* population was genetically distant from the other four populations, probably because the WS population was breeding on the Leizhou Peninsula, which was separated from the other four populations on Hainan Island by the Qiongzhou Strait and was the most geographically distant, so there was less gene flow with the other four populations, resulting in significant genetic differences.

### Population expansion pattern

In the present study, only the HW population of *Ae. aegypti* showed a significant negative Tajima’s D value, which is used to detect neutral deviation caused by population bottleneck, expansion, directed selection or infiltration [77]. The result may indicate a historical expansion of the HW population of *Ae. aegypti*. The YGH, HT and HW populations of *Ae. albopictus* may experience historical population expansion events based on the Fu's Fs values, which can be used to detect historical fluctuations in population size, and the value was more sensitive [78]. The result was consistent with the mismatch distribution (Fig. 4). The other two populations, BS and WS, had negative Tajima’s D and Fu’s F values, but these were not significant, suggesting that the expansion may have been restricted to separate local areas [79].

All the populations of the two *Aedes* species deviated significantly from HWE. Most showed a He > Ho result, except for the BS population of *Ae. albopictus*, indicating a deficit in heterozygosity, which may have been caused by environmental selective pressure [27, 80, 81]. The TPM model was considered the best fit to the microsatellite data, and the results indicated that the BS population of *Ae. aegypti* experienced a bottleneck, which may have been related to environmental changes and the competition from *Ae. albopictus*. Over the past 2 years, new ports have been built in Basuo, resulting in the destruction of *Ae. aegypti*'s breeding habitat and a sharp decline in species' numbers. At the same time, the local *Ae. albopictus* population has continued to invade its breeding sites, resulting in competition for space between the two species and ultimately leading to the decline of the *Ae. aegypti* population.

The Mantel test showed a significant positive correlation between genetic distance and geographic distance for the *Ae. aegypti* populations, but the correlation was not significant for *Ae. albopictus* populations, suggesting frequent gene flow between *Ae. albopictus* populations. This may be related to the rapid spread of these populations due to human activities, particularly the shipping trade [28]. Hainan Island has many trading ports in China, with cargo throughput reaching 199 million tons in 2020 [82]. The Qiongzhou Strait is an important transport link between Hainan Island and the Leizhou Peninsula, which is the only convenient gateway for maritime cargo between Hainan Island and the mainland. Frequent cargo trade inevitably leads to the continuous dispersal and gene exchange among *Ae. albopictus* populations in these areas.

## Conclusions

The present study assessed the genetic diversity of *Ae. aegypti* and *Ae. albopictus* from cohabiting fields on Hainan Island and the Leizhou Peninsula in China and, for the first time to our knowledge, explained the reasons for the sharp decline in *Ae. aegypti* populations on Hainan Island and the Leizhou Peninsula in terms of population genetics. The results suggested that the population diversity of *Ae. albopictus* was greater than that of *Ae. aegypti* and that *Ae. albopictus* had invaded *Ae. aegypti* breeding sites in some places and continued to spread on these islands. The active dispersal of *Ae. aegypti* was smaller than that of other mosquitoes, including the continuous niche invasion of *Ae. albopictus*, which may inevitably lead to the decline in the population numbers and genetic diversity of *Ae. aegypti*. Genetic diversity is used to measure the ability of a species to adapt to environmental change and to predict the direction of its future survival and development as well as to explore when and how it arose and to speculate on routes of migration and dispersal. Low genetic diversity in *Ae. aegypti* populations may indicate a decline in the mosquito's ability to adapt environmental changes. Therefore, the *Ae. aegypti* population is more likely to be controlled on Hainan Island and Leizhou Peninsula. In terms of future vector control, the most critical and effective measure is to control the spread of *Ae. albopictus* and monitor the population genetic dynamics of *Ae. aegypti*, which could theoretically support the further elimination of *Ae. aegypti* in Hainan Island and Leizhou Peninsula, China.

### Supplementary Information

**Additional file 1: Table S1.** Details on nine pairs and 11 pairs microsatellite loci of *Aedes aegypti* and *Ae. albopictus*, respectively.

**Additional file 2: Table S2.** Genetic relatedness in *Aedes aegypti* and *Ae. albopictus* populations by LRM.

**Additional file 3: Table S3.** Hardy-Weinberg equilibrium (HWE) based on nine microsatellite loci for *Aedes aegypti* and the 11 microsatellite loci for *Ae. albopictus*.

**Additional file 4: Table S4.** AMOVA based on different molecular markers for *Aedes aegypti* and *Ae. albopictus*.

**Additional file 5: Table S5.** Neutrality test for *Aedes aegypti* and *Ae. albopictus* based on *coxI* gene.

**Additional file 6: Table S6.** Fst and Nm matrix calculated for *Aedes aegypti* and *Ae. albopictus* based on *coxI* gene (Fst values below the diagonal and Nm (Nm = (1/Fst-1)/4) above the diagonal for every diagonal; bold numbers indicate significance at *P* < 0.05).

## Data Availability

The raw data supporting the conclusions of this article will be made available by the authors without undue reservation.

## Abbreviations

*coxI*: Mitochondrial cytochrome c oxidase subunit 1
PIC: Polymorphism information content
SI: Shannon’s diversity index
H: Number of haplotypes
Hd: Haplotype diversity
UPGMA: Unweighted pair-group method with arithmetic averaging nucleotide diversity
DAPC: Discriminant analysis of principal components
SSR: Simple sequence repeats
LRM: Lynch and Ritland estimator-mean
Na: Observed number of alleles
Ne: Effective number of alleles
Ho: Observed heterozygosity
He: Excepted heterozygosity
HWE: Hardy–Weinberg equilibrium
LD: Linkage disequilibrium
TPM: Two-phased mutation model
AMOVA: Analysis of molecular variation
Fst: Genetic differences among populations
Nm: Number of migrants
