# Supplementary material for: Genetic diversity of Aedes aegypti and Aedes albopictus from cohabiting fields in Hainan Island and the Leizhou Peninsula, China
Source: Parasit Vectors. 2023 Sep 8;16:319. doi: 10.1186/s13071-023-05936-5 (PMC10486073; doi:10.1186/s13071-023-05936-5)
Supplement: Supplementary file 1 — Additional file 1: Table S1. Details on nine pairs and 11 pairs microsatellite loci of Aedes aegypti and Ae. albopictus, respectively. [file 13071_2023_5936_MOESM1_ESM.docx]

**Table S1.** Details on 9 pairs and 11 pairs microsatellite loci of *Ae. aegypti* and *Ae. albopictus* respectively

| Mosquito | Loci | Primer Sequence (5’-3) | Repetitive Motifs | Expected Size（bp） |
| --- | --- | --- | --- | --- |
| *Ae. aegypti* | AT1 | F:CGTCGACGTTATCTCCTTGTT | AT | 156-174 |
|  |  | R:GGACCGGAAAGACACAGACA |  |  |
|  | AG2 | F:TCCCCTTTCAAACCTAATGG | AG | 115-178 |
|  |  | R:TTTGCCCTCGTATGCTCTCT |  |  |
|  | AG7 | F:CGTGCGAGTGAATGAGAGAC | GA | 153-185 |
|  |  | R:CATCCTCTCATCAGCTTCTAATAAA |  |  |
|  | AC2 | F:AATACAACGCGATCGACTCC | CA | 176-190 |
|  |  | R:AACGATTAGCTGCTCCGAAA |  |  |
|  | AC7 | F:TCGGCAAATTACCACAAACA | CA | 129-143 |
|  |  | R:CATTGGACTCGCTATAACACACA |  |  |
|  | B07 | F:CAAACAACGAACTGCTCACG | GA | 157-183 |
|  |  | R:TCGCAATTTCAACAGGTAGG |  |  |
|  | F06 | F:GCCAAAAACCAACAAACAGG | TAGA | 286-290 |
|  |  | R:AATCGACCCGACCAATAACA |  |  |
|  | SQM6 | F:CGACAGATGGTTACGGACGG | (TTTA)7(T)14 | 228 |
|  |  | R:GTCCCGCTCCAAAAATGCCC |  |  |
|  | SQM7 | F:AAAACCTGCGCAACAATCAT | AG4 | 147-169 |
|  |  | R:AAGGACTCCGTATAATCGCAAC |  |  |
| *Ae. albopictus* | BW-P1 | F:TTAGCATCCATCTATTCTGGC | (GT)6 | 230-260 |
|  |  | R:AAACATTCCTACGCATTTCAC |  |  |
|  | BW-P3 | F:GAAAATATGGTCTATCAAATG | (GT)3GC(GT)3T3 | 132-178 |
|  |  | R:AAGTCAGTAAAACAGGAGTCT |  |  |
|  | BW-P6 | F:GAATTGGGAGCTTGGTAAAAC | (TG)5 | 124-200 |
|  |  | R:CGCCTACTTGAGAAACACTGA |  |  |
|  | BW-P18 | F:CACTGGTTCTCTATCGAATGC | (GT)24 | 158-203 |
|  |  | R:GTGTTATCAGTTGGAAGCGTT |  |  |
|  | BW-P22 | F:GGCGTCCCCCCAACATACATC | (GT)5(GCGT)2(GT)3 | 187-244 |
|  |  | R:CAGCTCCGTCCTCCTCTTCCC |  |  |
|  | BW-P23 | F:GGATAAGAATGACACAGGCAC | (GAC)7 | 133-177 |
|  |  | R:CAAAGAGGAACACCATAGGAA |  |  |
|  | BW-P24 | F:ACGAAACATACTTACAATTGCA | (AC)8 | 145-239 |
|  |  | R:AACCTAGAGTCCGAGAGAGAAC |  |  |
|  | BW-P26 | F:CGTGGTGGTTAGGTCCATGTT | (GT)5 | 107-233 |
|  |  | R:TCGCTTTCGGCTCTAGTCAAT |  |  |
|  | BW-P27 | F:TTATACAAAAAGCGAACATCC | (ACG)6 | 249-281 |
|  |  | R:CACACACATAGAAAAAAGCAA |  |  |
|  | BW-P35 | F:TATTTGCACATCCATTTCGTCT | (CA)7T3 | 83-120 |
|  |  | R:TTCAAAACCTGATTTCCGACTG |  |  |
|  | BW-P36 | F:GTCATGTAGTCCTCACAGTCAC | (AC)6C3 | 159-179 |
|  |  | R:ATATGGATCATAGATGATGGAG |  |  |
