# Supplementary material for: Genetic diversity of Aedes aegypti and Aedes albopictus from cohabiting fields in Hainan Island and the Leizhou Peninsula, China
Source: Parasit Vectors. 2023 Sep 8;16:319. doi: 10.1186/s13071-023-05936-5 (PMC10486073; doi:10.1186/s13071-023-05936-5)
Supplement: Supplementary file 2 — Additional file 2: Table S2. Genetic relatedness in Aedes aegypti and Ae. albopictus populations by LRM. [file 13071_2023_5936_MOESM2_ESM.docx]

**Table S2.** Genetic relatedness in *Ae. aegypti* and *Ae. albopictus* populations by LRM

| Populations | *Ae. aegypti* | | | | *Ae. albopictus* | | | |
| --- | --- | --- | --- | --- | --- | --- | --- | --- |
|  | Between populations(N) | Percentage  (%) | Within populations(N) | Percentage  (%) | Between populations(N) | Percentage  (%) | Within populations(N) | Percentage  (%) |
| YGH | 7 | 0.04 | 31 | 0.18 | 6 | 0.06 | 3 | 0.03 |
| HW | 3 | 0.02 | 44 | 0.26 | 6 | 0.06 | 6 | 0.06 |
| WS | 0 | 0.00 | 36 | 0.21 | 9 | 0.09 | 9 | 0.09 |
| HT | 14 | 0.08 | 17 | 0.10 | 6 | 0.06 | 5 | 0.05 |
| BS | 7 | 0.04 | 55 | 0.32 | 44 | 0.42 | 44 | 0.42 |
| Total | 31 | 0.18 | 183 | 1.06 | 71 | 0.68 | 67 | 0.64 |
| Total pairwise comparisons 17205 | | | | | 10440 | | | |

N: number of comparisons
