# Supplementary material for: Genetic diversity of Aedes aegypti and Aedes albopictus from cohabiting fields in Hainan Island and the Leizhou Peninsula, China
Source: Parasit Vectors. 2023 Sep 8;16:319. doi: 10.1186/s13071-023-05936-5 (PMC10486073; doi:10.1186/s13071-023-05936-5)
Supplement: Supplementary file 3 — Additional file 3: Table S3. Hardy-Weinberg equilibrium (HWE) based on nine microsatellite loci for Aedes aegypti and the 11 microsatellite loci for Ae. albopictus. [file 13071_2023_5936_MOESM3_ESM.docx]

**Table S3.** Hardy–Weinberg equilibrium (HWE) based on 9 microsatellite loci for *Ae. aegypti* and the 11 microsatellite loci for *Ae. albopictus*

| Mosquito | locus | YGH | HT | BS | HW | WS |
| --- | --- | --- | --- | --- | --- | --- |
| *Ae. aegypti* | B07 | 0.1092^*^ | 0.2302^**^ | 0.0467^*^ | -0.0321 | 0.3428^***^ |
|  | F06 | 1^***^ | **/** | 1^*^ | **/** | 0.4316 |
|  | SQM6 | -0.0331 | -0.0243 | 0.1501 | 0.1277 | -0.0963 |
|  | SQM7 | 0.1135 | 0.1474^*^ | 0.1392 | 0.1079 | 0.1372^**^ |
|  | AT1 | -0.0238 | 0.0893 | -0.101 | -0.0861 | 0.1750^*^ |
|  | AG2 | 0.1172 | 0.0827 | 0.1079 | 0.3264^*^ | -0.0502^*^ |
|  | AG7 | 0.4744^***^ | 0.0031 | 0.3561^***^ | 0.2467^**^ | 0.4000^***^ |
|  | AC2 | 0.1830^*^ | -0.1502 | 0.0743 | -0.0093 | -0.0477 |
|  | AC7 | **/** | 0.0382 | -0.1724 | -0.0479 | 0.1154 |
| *Ae. albopictus* | BW-P1 | 0.4114**^***^** | 0.5623**^***^** | 0.7576**^***^** | 0.5302**^***^** | 0.5397**^***^** |
|  | BW-P3 | 0.6928**^*^** | 0.2930 | 0.1900 | 0.3854 | 0.7419**^*^** |
|  | BW-P6 | -0.2275 | 0.2100**^*^** | -0.2599 | -0.2535 | -0.4545 |
|  | BW-P18 | 0.1172^*^ | -0.1395 | 0.0323 | 0.2720**^**^** | -0.0189 |
|  | BW-P22 | 0.3817**^*^** | 0.1926 | 0.0233 | 0.2564**^*^** | 0.0112 |
|  | BW-P23 | 0.5623**^***^** | 0.1101 | -0.0871 | 0.4576**^***^** | 0.2965**^*^** |
|  | BW-P24 | -0.0065 | 0.3617**^***^** | 0.2000**^*^** | 0.1004 | 0.1336 |
|  | BW-P26 | -0.1464 | 0.1236 | 0.0317 | -0.0642 | -0.1139 |
|  | BW-P27 | 0.5917**^***^** | 0.7492**^***^** | 0.4476**^***^** | 0.4195**^***^** | 0.4811**^*^** |
|  | BW-P35 | 0.2427 | 0.1056 | 0.5566**^***^** | 0.2584**^***^** | -0.1803 |
|  | BW-P36 | 0.4927**^***^** | 0.1342 | 0.5033**^***^** | 0.5260**^***^** | 0.0734 |

* P<0.05, ** P<0.01, *** P<0.001
