# Supplementary material for: Genetic diversity of Aedes aegypti and Aedes albopictus from cohabiting fields in Hainan Island and the Leizhou Peninsula, China
Source: Parasit Vectors. 2023 Sep 8;16:319. doi: 10.1186/s13071-023-05936-5 (PMC10486073; doi:10.1186/s13071-023-05936-5)
Supplement: Supplementary file 4 — Additional file 4: Table S4. AMOVA based on different molecular markers for Aedes aegypti and Ae. albopictus. [file 13071_2023_5936_MOESM4_ESM.docx]

**Table S4.** AMOVA based on different molecular markers for *Ae. aegypti* and *Ae. albopictus*

| Molecular Marker | Mosquito | Source of variation | df | Sum of squares | Variance components | Percentage of variation (%) | Fst |
| --- | --- | --- | --- | --- | --- | --- | --- |
| SSR | *Ae. aegypti* | Among populations | 4 | 78.944 | 0.29463 | 9.34 |  |
|  |  | Within populations | 275 | 784.038 | 2.86053 | 90.66 |  |
|  |  | Total | 279 | 862.982 | 3.15516 | 100.00 | 0.09338^*^ |
|  | *Ae. albopictus* | Among populations | 4 | 53.677 | 0.15678 | 3.58 |  |
|  |  | Within populations | 275 | 1070.253 | 4.22017 | 96.42 |  |
|  |  | Total | 279 | 1123.920 | 4.37695 | 100 | 0.03600^*^ |
| *coxI* | *Ae. aegypti* | Among populations | 4 | 30.430 | 0.22893 | 15.44 |  |
|  |  | Within populations | 135 | 169.270 | 1.25385 | 84.56 |  |
|  |  | Total | 139 | 199.700 | 1.48278 | 100.00 | 0.15439^*^ |
|  | *Ae. albopictus* | Among populations | 4 | 19.265 | 0.12207 | 12.21 |  |
|  |  | Within populations | 125 | 132.119 | 1.05695 | 87.79 |  |
|  |  | Total | 129 | 151.385 | 1.20392 | 100.00 | 0.12207^*^ |

* P<0.05
