# Supplementary material for: Genetic diversity of Aedes aegypti and Aedes albopictus from cohabiting fields in Hainan Island and the Leizhou Peninsula, China
Source: Parasit Vectors. 2023 Sep 8;16:319. doi: 10.1186/s13071-023-05936-5 (PMC10486073; doi:10.1186/s13071-023-05936-5)
Supplement: Supplementary file 5 — Additional file 5: Table S5. Neutrality test for Aedes aegypti and Ae. albopictus based on coxI gene. [file 13071_2023_5936_MOESM5_ESM.docx]

**Table S5.** Neutrality test for *Ae. aegypti* and *Ae. albopictus* based on *coxI* gene

| Mosquito | Population | N | Nh^a^ | Tajima’D | P^b^ | Fu’s Fs | P^b^ |
| --- | --- | --- | --- | --- | --- | --- | --- |
| *Ae. aegypti* | YGH | 30 | 6 | -0.35199 | 0.42200 | -2.07021 | 0.08300 |
|  | HT | 31 | 5 | 0.55794 | 0.73100 | 0.52246 | 0.62800 |
|  | BS | 18 | 4 | 0.47800 | 0.70900 | 1.12247 | 0.74000 |
|  | HW | 28 | 6 | -1.66551 | 0.03500* | 2.14678 | 0.84200 |
|  | WS | 33 | 8 | -0.54362 | 0.34600 | 1.86595 | 0.79800 |
| *Ae. albopictus* | YGH | 30 | 14 | -1.12997 | 0.14000 | -7.82205 | 0.00100* |
|  | HT | 30 | 9 | -1.31245 | 0.09300 | -3.44726 | 0.02300* |
|  | BS | 27 | 8 | -0.38703 | 0.38000 | -1.83302 | 0.13300 |
|  | HW | 30 | 15 | -1.62321 | 0.03800* | -8.50950 | 0.00000* |
|  | WS | 13 | 8 | -0.06561 | 0.48000 | -1.75037 | 0.14600 |

a: number of haplotypes; b: * indicated P<0.05.
