# Supplementary material for: Genetic diversity of Aedes aegypti and Aedes albopictus from cohabiting fields in Hainan Island and the Leizhou Peninsula, China
Source: Parasit Vectors. 2023 Sep 8;16:319. doi: 10.1186/s13071-023-05936-5 (PMC10486073; doi:10.1186/s13071-023-05936-5)
Supplement: Supplementary file 6 — Additional file 6: Table S6. Fst and Nm matrix calculated for Aedes aegypti and Ae. albopictus based on coxI gene (Fst values below the diagonal and Nm (Nm = (1/Fst-1)/4) above the diagonal for every diagonal; bold numbers indicate significance at P < 0.05). [file 13071_2023_5936_MOESM6_ESM.docx]

**Table S6.** Fst and Nm matrix calculated for *Ae. aegypti* and *Ae. albopictus* based on *coxI* gene (Fst values below the diagonal and Nm (Nm = (1/ Fst-1)/4) above the diagonal for every diagonal, asterisked numbers indicated significant at p<0.05)

| *Ae. aegypti* | WS | HW | BS | HT | YGH |
| --- | --- | --- | --- | --- | --- |
| WS |  | 1.12961 | 1.65302 | 1.29895 | 1.52936 |
| HW | 0.18121* |  | 0.98561 | 2.96295 | 1.21379 |
| BS | 0.13137* | 0.20233* |  | 1.03694 | 1.23236 |
| HT | 0.16140* | 0.07781* | 0.19426* |  | 2.58543 |
| YGH | 0.14050* | 0.17079* | 0.16865* | 0.08817* |  |
| *Ae. albopictus* | WS | HW | BS | HT | YGH |
| WS |  | 0.75337 | 0.69514 | 0.49587 | 1.10077 |
| HW | 0.24916* |  | 8.11960 | 13.73210 | 3.72962 |
| BS | 0.26451* | 0.02987 |  | 6.54347 | 2.43528 |
| HT | 0.33518* | 0.01788 | 0.03680* |  | 1.73776 |
| YGH | 0.18508* | 0.06282* | 0.09310* | 0.12577* |  |
